# Immunosuppressive Properties of Epidermal Keratinocytes Differ According to Their Immaturity Status

**DOI:** 10.3389/fimmu.2022.786859

**Published:** 2022-02-11

**Authors:** Guillaume Mestrallet, Edgardo D. Carosella, Michele T. Martin, Nathalie Rouas-Freiss, Nicolas O. Fortunel, Joel LeMaoult

**Affiliations:** ^1^ Commissariat à l’Energie Atomique et aux Energies Alternatives (CEA), DRF, Francois Jacob Institute of Biology, Laboratory of Genomics and Radiobiology of Keratinopoiesis, Institute of Cellular and Molecular Radiobiology, Evry, France; ^2^ Université Paris-Saclay, Saint-Aubin, France; ^3^ Commissariat à l’Energie Atomique et aux Energies Alternatives (CEA), DRF, Francois Jacob Institute of Biology, Hemato-Immunology Research Department, Saint-Louis Hospital, Paris, France; ^4^ U976 HIPI Unit, IRSL, Université Paris, Paris, France

**Keywords:** immunosuppression, keratinocyte stem cells, HLA-G, PD-L1, CD4^+^ T-cell proliferation

## Abstract

Preservation of a functional keratinocyte stem cell pool is essential to ensure the long-term maintenance of epidermis integrity, through continuous physiological renewal and regeneration in case of injury. Protecting stem cells from inflammation and immune reactions is thus a critical issue that needs to be explored. Here, we show that the immature CD49f^high^ precursor cell fraction from interfollicular epidermis keratinocytes, comprising stem cells and progenitors, is able to inhibit CD4**^+^** T-cell proliferation. Of note, both the stem cell-enriched CD49f^high^/EGFR^low^ subpopulation and the less immature CD49f^high^/EGFR^high^ progenitors ensure this effect. Moreover, we show that HLA-G and PD-L1 immune checkpoints are overexpressed in CD49f^high^ precursors, as compared to CD49f^low^ differentiated keratinocytes. This potency may limit immune reactions against immature precursors including stem cells, and protect them from exacerbated inflammation. Further exploring this correlation between immuno-modulation and immaturity may open perspectives in allogenic cell therapies.

## Introduction

Stem cells are essential for the maintenance and renewal of tissues, thus survival of this cellular pool is critical (1). Here, we explored the question whether the degree of keratinocyte ‘stemness’ in the interfollicular epidermis is associated with a particular immune status through expression of immune checkpoints. Understanding the immune properties of these adult stem cells will provide information on the mechanisms protecting these cells from immune reactions, and contributing to the maintenance of skin tissue in a stress environment.

Human epidermal keratinocytes can be fractionated into functional subpopulations according to cell-surface phenotypes. Basal keratinocytes can be first selected from the total keratinocyte population based on high integrin α6 (CD49f) expression (2) or rapid adhesion on collagen (3). Then subpopulations enriched in stem or progenitor cells can be separated according to cell surface receptors. A first described phenotype used the level of transferrin receptor (TFR) to distinguish quiescent stem cells (TFR^low^) from cycling progenitors (TFR^high^) (2). The present work used the EGF receptor (EGFR) for this purpose (3). The most immature [stem cell-enriched] subpopulation (EGFR^low^) was functionally characterized as endowed with a higher long-term growth and epidermis reconstruction potential, as compared to that of the less immature [progenitor-enriched] subpopulation (EGFR^high^) which potential declined after short-term growth (3).

Several molecular effectors have been involved in the maintenance or loss of the stem cell state in human keratinocytes, including the transcription factor KLF4, whose expression level is critical for this cellular fate (4). More generally, a complex gene network has been linked to the regulation of stemness in human keratinocytes, involving shutdown of differentiation signals together with induction of self-renewal-promoting effectors (5). Of note, the reprogramming of human keratinocytes into iPSCs through vector-driven expression of the transcription factors KLF4, cMYC, OCT3/4 and SOX2 constituted a good model of stemness promotion (6).

Epidermis regeneration involves both immune and cell renewal properties. For example, the stemness gene *KLF4* also regulates anti-inflammatory genes in murine keratinocytes *via* an interaction with the glucocorticoid receptor (GR) (7). This regulation operates *via* the mediators of inflammation Tsc22d3 and Zfp36. The interaction between KLF4 and GR therefore plays on the balance between keratinocyte differentiation *versus* proliferation. In mice, in case of skin injury altering intercellular junctions, resident epithelial stem cells also modify their gene expression profile and recruit immune cells (DCs and Treg cells) that will in turn stimulate the proliferation of stem cells (8).

In human skin, we have recently demonstrated that keratinocytes from interfollicular epidermis inhibit allogenic CD4^+^ T-cell proliferation, notably through the secretion of TGFB1 and the cell-surface expression of HLA-G1 and PD-L1 immune checkpoints (9). Several precursor cells, such as mesenchymal stem cells and hair follicle stem cells (10), have developed immune escape mechanisms and exhibit immunosuppressive properties likely to help ensure their survival in deleterious conditions. The present work aimed at investigating such immunosuppressive mechanisms in keratinocyte precursors of the human interfollicular epidermis. Studies in a mouse model have shown that keratinocytes expressing the immune checkpoint PD-L1 reduce the proliferation and effector function of T cells at inflammatory sites (11). Notably, PD-L1 expression was reduced in human psoriatic epidermis, which could promote the activity of effector T cells (12). PD-L1 binds to PD-1, expressed on the surface of T cells, inhibits their activity (13) and limits autoimmune reactions (14). The non-classical HLA class I molecule HLA-G is another immune checkpoint molecule originally described as permitting maternal-fetal tolerance (15). Notably, transplanted patients expressing HLA-G were significantly less prone to acute and chronic transplant rejection in solid organ transplantation such as in heart, kidney, liver and lung transplantation (16–19). HLA-G inhibits the cytolytic function of NK and T cells, the alloproliferative response of CD4^+^ T cells, the antibody production by B cells and the antigen-presenting function of dendritic cells (20). It also induces the emergence of regulatory cells such as Tregs and MDSC. In cancer, HLA-G and PD-L1 expressed by tumor cells have been shown to inhibit different populations of T cells (21, 22).

The present work aimed at investigating whether keratinocyte stem and progenitor cells from the human interfollicular epidermis develop specific immunosuppressive properties by modifying the expression of HLA-G1 and PD-L1 immune checkpoints.

## Materials and Methods

### Human Tissues and Cells

The present study was approved by the review board of the iRCM (Institut de Radiobiologie Cellulaire et Moléculaire, CEA (Atomic Energy Commission), Fontenay-aux-Roses, France), and is in accordance with the scientific, ethical, safety and publication policy of the CEA (CODECO number DC-2008-228, reviewed by the ethical research committee IDF-3). PBMCs were collected from healthy donors in the French Blood Bank (EFS) at Saint Louis Hospital (Paris) after informed consent was obtained. Human skin tissue from healthy adult donors was collected in the context of breast reduction surgery, after obtaining informed consent. Epidermal keratinocytes and dermal fibroblasts were extracted as previously described (4). Briefly, enzymatic treatment with a solution containing (v/v) 3/4 grade II dispase 2.4 U mL−1 (Roche Molecular Biochemicals, Mannheim, Germany) and 1/4 trypsin 0.25% (Gibco) was conducted for 24 h at 4°C. We used two categories of cells in this study: cells directly extracted from the tissue and not amplified (tissue keratinocytes), and cells extracted from the tissue, amplified and used between passage 1 and 3 in culture (amplified keratinocytes).

### Cell Culture

Amplified adult epidermal keratinocytes were obtained as follows: bulk cultures were generated in a serum-containing medium, in the presence of a feeder layer of human dermal fibroblasts growth-arrested by 60 Gy γ irradiation (23). All cultures were performed in plastic flasks coated with type-I collagen (BioCoat, Becton-Dickinson, Le Pont de Claix, France). The composition of the serum-containing medium consisted of DMEM and Ham’s F12 media (Gibco, ThermoFisher, Les Ulis, France) (v/v, 3/1 mixture), 10% fetal calf serum (Hyclone, Fisher Scientific, Illkirch, France), 10 ng/mL epidermal growth factor (EGF) (Chemicon, Fisher Scientific, Illkirch, France), 5 μg/mL transferrin (Sigma, Saint-Quentin Fallavier, France), 5 μg/mL insulin (Sigma, Saint-Quentin Fallavier, France), 0.4 μg/mL hydrocortisone (Sigma, Saint-Quentin Fallavier, France), 180 μM adenine (Sigma, Saint-Quentin Fallavier, France), 2 mM tri-iodothyronine (Sigma, Saint-Quentin Fallavier, France), 2 mM L-glutamine (Gibco, ThermoFisher, Les Ulis, France), and 100 U/mL penicillin/streptomycin (Gibco, ThermoFisher, Les Ulis, France). The medium was renewed 3 times a week. For cell amplification, keratinocytes were seeded at 1000 cells/cm2 and sub-cultured weekly. Feeder cells were seeded at 5000 cells/cm2.

### Flow Cytometry Analysis

For analysis of cell-surface immune marker expression, keratinocytes were processed as single-cell suspensions and stained for 1 h at room temperature with monoclonal antibodies. The staining monoclonal antibodies used were: phycoerythrin (PE) conjugated rat anti-human CD49f (ITA6) (clone GoH3, BD Pharmingen, Le Pont de Claix, France), Alexa Fluor 405 conjugated rat anti-human EGFR (clone ICR10, Novus Biologicals, Lille, France), PE-cy7-conjugated mouse anti-human PD-L1 (clone MIH1, Thermo Fisher, Les Ulis, France), Alexa 700-conjugated mouse anti-human HLA-G (clone 87G, Novus Biologicals, Lille, France), FITC conjugated mouse anti-human TGFB (clone 1D11, R&D Systems), PE conjugated mouse anti-human IL-10 (clone B-S10, Diaclone). Non-reactive antibodies of similar species and isotype, coupled with the same fluorochromes, were used as isotypic controls. CD49f, EGFR, PD-L1, HLA-G, TGFB and IL-10 expression profiles were analyzed using an Astrios cell-sorter (Beckman Coulter, Villepinte, France) or Attune NxT (Thermo Fisher, Les Ulis, France) or MACSquant (Miltenyi, Paris, France) analyzer. Data were analyzed using FlowJo software (BD Biosciences, Le Pont de Claix, France).

### Cell Sorting

Adult epidermal keratinocytes were sorted according to CD49f and EGFR expression, using phycoerythrin (PE)-conjugated rat anti-human CD49f (ITA6) monoclonal antibody (clone GoH3, BD Pharmingen, Le Pont de Claix, France) and Alexa Fluor 405 conjugated rat anti-human EGFR monoclonal antibody (clone ICR10, Novus Biologicals, Lille, France). Appropriate isotype controls were systematically used. Cells were sorted using a FACS Aria 3 sorter (BD Biosciences, Le Pont de Claix, France).

### High-Content Imaging and Screening

Keratinocytes were plated in 96 well plates (TPP, Trasadingen, Switzerland) at a concentration of 3000 cells/cm^2^. After 1 week of growth, cells were stained for 1 hour at room temperature using the following monoclonal antibodies: phycoerythrin (PE)-conjugated rat anti-human CD49f (ITA6) (clone GoH3, BD Pharmingen, Le Pont de Claix, France), APC-conjugated mouse anti-human PD-L1 (clone MIH1, Thermofisher, Les Ulis, France), Alexa700-conjugated mouse anti-human HLA-G (clone 87G, Novus Biologicals, Lille, France). Appropriate isotype controls were systematically used. Nuclei staining was performed using Hoescht (Thermofisher, Les Ulis, France). CD49f, PD-L1, CD40, MHC1 and HLA-G expression profiles were analyzed using AnalysisCellInsight CX7 High-Content Screening (HCS) Platform (Thermofisher, Les Ulis, France).

### Flow Cytometry-Based Analysis of CD4^+^ T-Cell Proliferation

Keratinocytes were seeded at various ratios in 96-well culture plates (collagen-1 96-well, BD BioCoat, Le Pont de Claix, France) and incubated for 4 h at 37°C, in 5% CO2. Then, PBMCs were incubated for 1 h at 37°C, in 5% CO2 in 100 μL RPMI medium (Sigma, Saint-Quentin Fallavier, France) supplemented with 20% FCS, enriched in streptomycin and glucose. PBMCs were incubated for 20 min with a proliferation dye (eBioscience Cell Proliferation Dye eFluor 450, Thermo Fisher, Les Ulis, France), then stimulated or not by anti-CD2:anti-CD3:anti-CD28-coated beads (T-Cell Activation/Expansion Kit, Miltenyi, Paris, France, one bead per cell), and seeded at 100,000 cells per well with or without keratinocytes. PBMC proliferation was quantified by reduction in cell dye intensity after 7 days. Analysis was performed on day 7, using a flow cytometer. The ability of keratinocytes to modulate CD4^+^ T-cell proliferation was analyzed by comparing CD4^+^ T-cell dye intensity decrease in the presence versus absence of keratinocytes. For this, cells were stained for 20 minutes at room temperature using Viobright FITC conjugated mouse anti-human CD4 (clone REA623, Miltenyi).

### Statistics

Significant differences were assessed *via* 2-tailed Mann–Whitney U-test or t-test. All data are presented as mean ± SEM. Differences were considered significant for p < 0.05; * = p < 0.05, ** = p < 0.01, *** = p < 0.001 and **** = p < 0.0001.

## Results

### Tissue Keratinocyte Precursors Inhibit CD4^+^ T-Cell Proliferation

**Figure 1:**
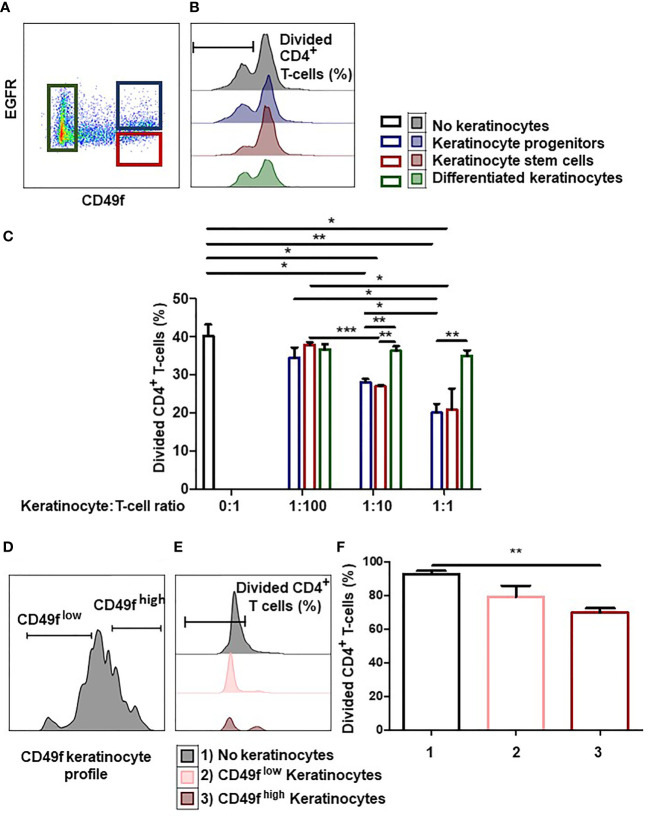
Keratinocyte precursors limit CD4^+^ T cell proliferation. **(A)** Representative flow cytometry profiles of keratinocytes directly extracted from the tissue and sorted according to their immaturity level visualized thanks to CD49f and EGFR stainings. **(B, C)** 1,000, 10,000 or 100,000 tissue keratinocytes (adult stem cells, progenitors or differentiated cells) from one representative donor were incubated with 100,000 PBMC for 7 days. PBMCs were pre-marked with a proliferation dye. PBMC were activated by CD3**^+^** CD28**^+^** beads. PBMC proliferation was quantified by dye decrease at day 7. **(B)** Representative flow cytometry profiles at day 7. **(C)** CD4**^+^** T-cell proliferation inhibition according to the keratinocyte number and immaturity level (mean ± SEM, p<0.05, n=3). **(D)** Representative flow cytometry profiles of amplified keratinocytes sorted according to their CD49f level. **(E, F)** 10,000 amplified keratinocytes, sorted according to their CD49f expression after amplification for 7 days, were incubated with 100,000 PBMC during 7 days. PBMCs were pre-marked with a dye. PBMC were activated by CD3**^+^** CD28**^+^** beads. PBMC proliferation was quantified by dye decrease at day 7. **(E)** Representative flow cytometry profiles at day 7. F CD4**^+^** T-cell proliferation inhibition according to the keratinocyte CD49f expression (mean ± SEM, p<0.05, n=3). Exact p-values were determined according to the t-test * = p < 0.05, ** = p < 0.01 and *** = p < 0.001.

We investigated whether the degree of immaturity of keratinocytes modulates their immunomodulatory properties. To this end, we analyzed whether adult keratinocytes at three differentiation stages (stem cells, progenitors, or differentiated) could differentially inhibit CD4**^+^** T-cell proliferation. For this, tissue keratinocytes were sorted by flow cytometry according to their immaturity level (**Figure 1A**). Keratinocyte stem cells were defined as CD49f^high^ EGFR^low^, keratinocyte progenitor cells as CD49f^high^ EGFR^high^, and differentiated keratinocytes as CD49f^low^. Isotypic controls for CD49f and EGFR are shown. Sorted cells were then incubated with activated PBMC pre-labeled with a proliferation dye (**Figure 1B**). We observed differences in CD4**^+^** T-cell proliferation inhibition according to the keratinocyte immaturity level (mean ± SEM, p<0.05, n=3) (**Figure 1C**). As can be seen, keratinocyte stem cells and progenitors (i.e. keratinocyte precursors) inhibited CD4**^+^** T-cell proliferation at low numbers, whereas differentiated keratinocytes did not. This CD4**^+^** T-cell proliferation inhibition increased in proportion to the number of keratinocyte precursors present (**Figure 1C**). We noticed that no effect was observed in the condition with differentiated keratinocytes, contrarily to conditions with immature keratinocytes, although responder cells and beads were the same in every condition. This result rules out steric hindrance as a mechanism for responder cell proliferation inhibition. Tissue keratinocyte precursors were therefore more effective than differentiated keratinocytes in suppressing CD4^+^ T-cell proliferation.

Next, we determined whether keratinocyte precursors still inhibited CD4**^+^** T cell-proliferation after *in vitro* amplification. For this, we cultured tissue keratinocytes *in vitro* for 1 to 3 weeks (1 passage per week), and sorted CD49f^high^ keratinocyte precursors (stem + progenitors) and CD49f^low^ differentiated keratinocytes (2). (**Figure 1D**). Sorted keratinocytes were then incubated with activated PBMC pre-labeled with a proliferation dye (**Figure 1E**). We observed that only keratinocyte precursors suppressed CD4**^+^** T-cell proliferation (mean ± SEM, p<0.05, n=3) (**Figure 1F**). Keratinocyte precursors therefore retained their ability to reduce CD4**^+^** T-cell proliferation after *in vitro* amplification.

### Keratinocyte Precursors Overexpress the Immune Checkpoints HLA-G and PD-L1

**Figure 2:**
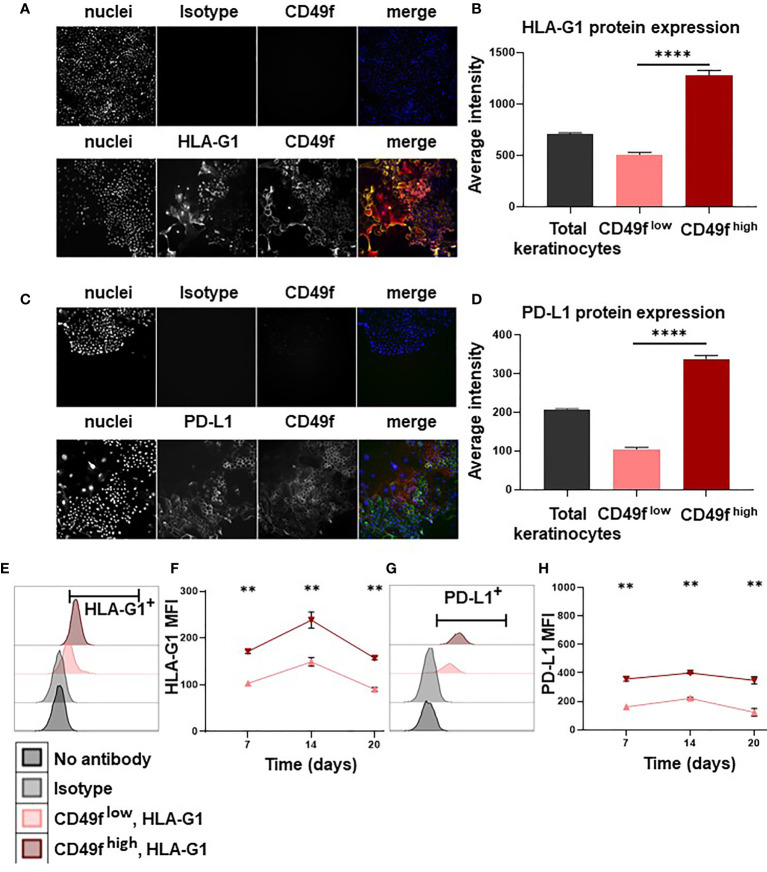
Keratinocyte precursors overexpress the immune checkpoints HLA-G1 and PD-L1. Cells from one representative donor were cultivated for 7, 14 or 20 days in a culture medium with serum and a layer of feeder cells. Analysis by high content single-cell image analysis. **(A, C)** Representative profiles of HLA-G1 and PD-L1 expression according to CD49f expression on keratinocytes amplified for 7 days. **(B, D)** High content single-cell image analysis of HLA-G1 and PD-L1 levels (Average intensity of fluorescence) (mean ± SEM, p<0.0001, n=1,000 cells*3 culture replicate). **(E, G)** Representative flow cytometry profiles of HLA-G1 and PD-L1 expression on keratinocytes amplified for 7 days. **(F, H)** Analysis by flow cytometry of HLA-G1 and PD-L1 expression (mean ± SEM, n=6) according to CD49f level on keratinocytes amplified for 7/14/20 days. Exact p-values were determined according to the Mann-Whitney U-test ** = p < 0.01 and **** = p < 0.0001.

We then investigated the mechanisms by which keratinocyte precursors inhibit CD4^+^ T-cell proliferation. By high content single-cell image analysis, we observed that 7-days amplified keratinocytes expressed HLA-G and PD-L1 immune checkpoints (**Figures 2A, C**). When HLA-G and PD-L1 were analyzed according to CD49f expression, we found that amplified keratinocytes that had the highest levels of CD49f overexpressed both HLA-G and PD-L1 (mean ± SEM, p<0.0001, n=3) (**Figures 2B, D**). These data were confirmed by flow cytometry (**Figures 2E, G**). Furthermore, keratinocytes were analyzed for expression of the immune suppressive cytokines IL-10 and TGFB. No IL-10 expression was observed. TGFB expression was found in all keratinocyte subpopulations, but no difference in expression levels was observed. (**Supplementary Figure 1**). This phenotype was stable, as CD49f^high^ keratinocytes that had been amplified for 3 weeks (1 passage per week) still displayed the highest expression levels of HLA-G and PD-L1 (mean ± SEM, n=6) (**Figures 2F, H**).

## Discussion

Keratinocytes obtained from donor skin samples could be separated into stem cells with an CD49f^high^ EGFR^low^ phenotype, progenitors with an CD49f^high^ EGFR^high^ phenotype, or differentiated keratinocytes with an CD49f^low^ phenotype (2, 3). We show here that these populations exhibited distinct immune profiles. Compared to differentiated keratinocytes, progenitors and stem cells, whether tissue extracted or amplified in cell culture, exert immunomodulatory properties by inhibiting CD4**^+^** T-cell *in vitro* proliferation.

We have recently shown that human keratinocytes inhibit allogenic CD4**^+^** T-cell proliferation through secretion of soluble factors and cell-surface expression of HLA-G and PD-L1 immune checkpoints (9). In the present study, we refine these results by considering keratinocyte populations according to their level of immaturity, and show that keratinocyte precursors with high levels of cell-surface CD49f overexpress HLA-G and PD-L1. Studies in mice indicated that keratinocytes expressing PD-L1 reduced the proliferation and effector function of T cells at local inflammatory sites (11). PD-L1 expression also correlated with a higher presence of Tregs in the mice skin (24). PD-L1 binds to PD-1 expressed on the surface of T cells, inhibits their activity (13), and limits autoimmune reactions (14). HLA-G inhibits the cytolytic function of NK and T cells, the alloproliferative response of CD4**^+^** T-cells, the antibody production by B cells and the antigen-presenting function of dendritic cells (20, 25). Notably, HLA-G expression was also described in other immature cell types, such as mesenchymal stem cells, osteoblasts, chondroblasts, erythroid progenitors and hepatic stem cells (26). It has been proposed that stem cells are preserved from immune attacks, a phenomenon called immune privilege (10). This is in accordance with a recent study demonstrating that, unlike mature endothelial cells, their progenitors [Endothelial progenitor cells (EPCs) or Endothelial Colony-Forming Cells (ECFCs)] were immunosuppressive and that this was linked with HLA-G, IL-10 and TGFB expression (27, 28). Overexpression of PD-L1 and HLA-G on keratinocyte precursors may support this immune privilege by protecting them against a prolonged abnormal immune response, such as in auto-immunity and chronic inflammation (9).

In this regard, murine hair follicle stem cells downregulate Nlrc5 and MHCI in their quiescent state (10). Expression of Nlrc5 upregulates MHC1 on murine hair follicle stem cells, so its downregulation explains that of the MHC1. The alteration of this immune privilege is one of the causes of alopecia areata (29). This pathological context was described as an autoimmune disorder, in which an abnormal infiltration of T cells causes local inflammation and destruction of anagen hair follicles (30). Expression of MHC by keratinocytes promotes the maintenance of autoreactive T cells directed against hair follicles (31). In psoriasis, another autoimmune pathology, epidermal cells are renewed every 3 to 5 days rather than 28 to 30 days in normal conditions (32). It would be interesting to investigate if this high renewal is linked to immune checkpoints expression, knowing that HLA-G and PD-L1 are expressed in psoriatic skin (33, 34). Investigating the expression of immune checkpoints on keratinocyte precursors in hair follicles could therefore provide a better understanding of their immune privilege and autoimmune-associated pathologies.

The immunosuppressive properties exerted by keratinocyte progenitors may also be involved in the development of skin cancer. Adult keratinocyte stem cells can drift into cancer cells, leading to cutaneous squamous cell carcinoma or basal cell carcinoma development (35). Tumor growth is known to be enhanced by cancer cell ability to escape elimination by the immune system (36). HLA-G and PD-L1 inhibit different populations of T cells in cancer (21, 22), and therefore critically contribute to tumor escape from immunosurveillance. PD-L1 expression and targeting were particularly well documented in squamous cell carcinoma (37). As immunotherapies are increasingly used to direct the body’s immune system against tumor (38), overexpression of PD-L1 and HLA-G by keratinocyte precursors should be considered in understanding the function of skin tumor initiator cells.

Finally, the immunosuppressive properties of keratinocyte precursors could be beneficial for skin repair therapy. Human keratinocytes derived from the hair follicle can promote healing (39) or be used to treat leg ulcers (40). We postulate that the use of keratinocyte precursors from the interfollicular epidermis, engineered to express high levels of HLA-G and PD-L1, could promote skin graft tolerance and open perspectives for their use in allogeneic settings for cell therapy (9). Indeed, the generation of allogeneic skin grafts presents a double stake. On the one hand, the maintenance of epidermal stem cells with high regenerative potential, and on the other hand, obtaining an immunocompatible graft.

## Data Availability Statement

The raw data supporting the conclusions of this article will be made available by the authors, without undue reservation.

## Ethics Statement

The studies involving human participants were reviewed and approved by ethical research committee IDF-3. The patients/participants provided their written informed consent to participate in this study.

## Author Contributions

GM designed the study, performed experiments, analyzed data, wrote the manuscript. EDC, NR-F, MM, NF, and JL designed the study, analyzed data, wrote the manuscript. All authors contributed to the article and approved the submitted version.

## Funding

This research received no external funding. It was supported by CEA funds, including a CFR Ph.D. program grant (2018-2021).

## Conflict of Interest

The authors declare that the research was conducted in the absence of any commercial or financial relationships that could be construed as a potential conflict of interest.

## Publisher’s Note

All claims expressed in this article are solely those of the authors and do not necessarily represent those of their affiliated organizations, or those of the publisher, the editors and the reviewers. Any product that may be evaluated in this article, or claim that may be made by its manufacturer, is not guaranteed or endorsed by the publisher.

## Supplementary Material

Supplementary Figure 1 Expression of immunomodulatory cytokines by keratinocyte precursors. Analysis by flow cytometry. **(A)** Expression of IL-10 by keratinocytes. **(B)** Expression of TGFB by keratinocytes according to their immaturity level (mean ± SEM, n=6). Exact p-values were determined according to the Mann-Whitney U-test.

## Abbreviations

CD49f, Integrin α6 (ITA6); EGFR, EGF receptor; KLF4, Kruppel-like factor 4; MHC, major histocompatibility complex; HLA-G, human leucocyte antigen G; PD-L1, programmed death-ligand 1.
