# Supplementary material for: Immunosuppressive Properties of Epidermal Keratinocytes Differ According to Their Immaturity Status
Source: Front Immunol. 2022 Feb 11;13:786859. doi: 10.3389/fimmu.2022.786859 (PMC8878806; doi:10.3389/fimmu.2022.786859)
Supplement: Supplementary Figure 1 — Expression of immunomodulatory cytokines by keratinocyte precursors. Analysis by flow cytometry. (A) Expression of IL-10 by keratinocytes. (B) Expression of TGFB by keratinocytes according to their immaturity level (mean ± SEM, n=6). Exact p-values were determined according to the Mann-Whitney U-test. [file Presentation_1.pptx]

## Slide 1
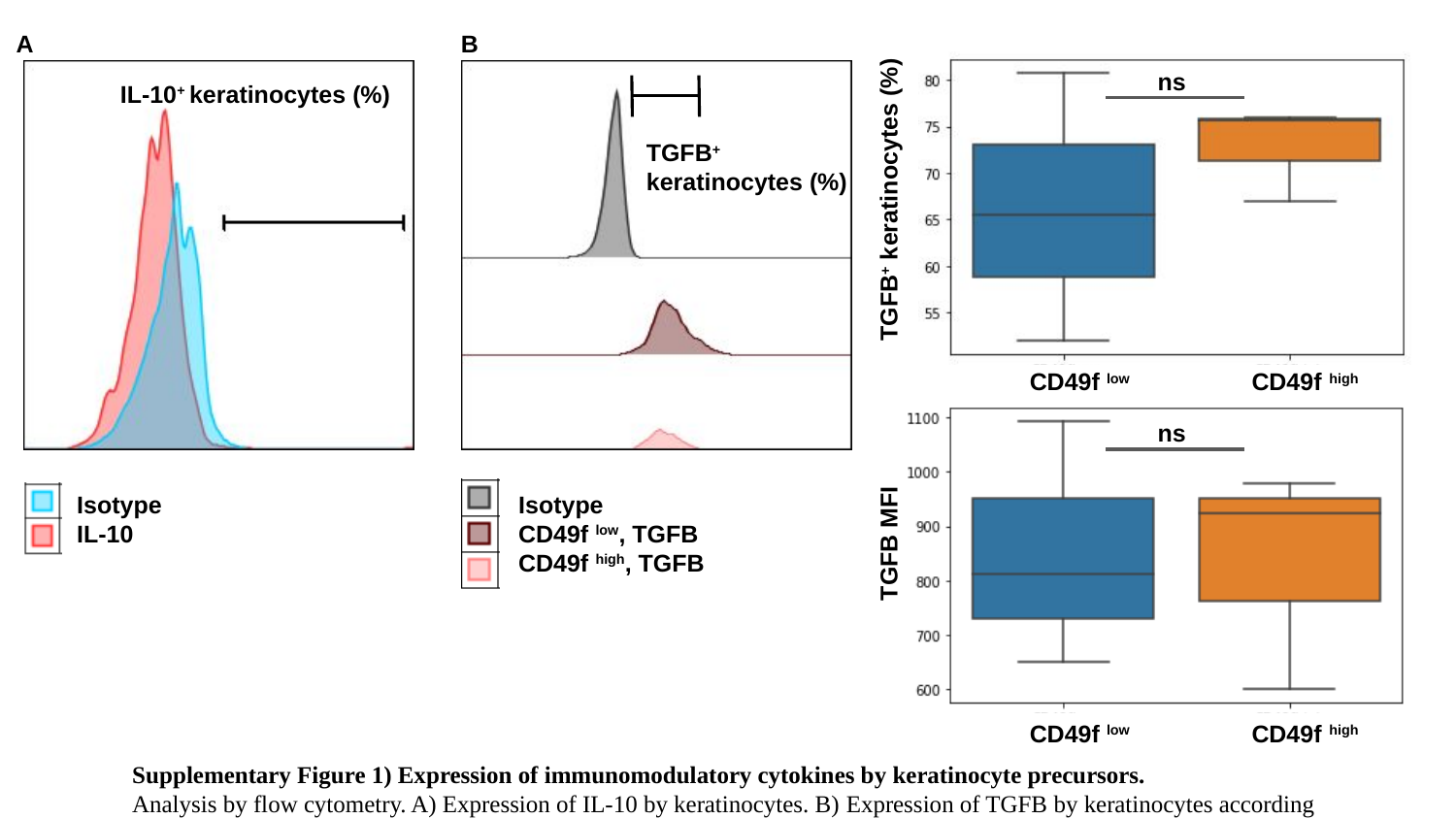

A
B
ns
IL-10+ keratinocytes (%)
TGFB+ keratinocytes (%)
TGFB+ keratinocytes (%)
CD49f low CD49f high
ns
Isotype
IL-10
Isotype
CD49f low, TGFB
CD49f high, TGFB
TGFB MFI
CD49f low CD49f high
Supplementary Figure 1) Expression of immunomodulatory cytokines by keratinocyte precursors.
Analysis by flow cytometry. A) Expression of IL-10 by keratinocytes. B) Expression of TGFB by keratinocytes according to their immaturity level (mean±SEM, n=6). Exact p-values were determined according to the Mann-Whitney U-test.
